# Acupuncture and Moxibustion for Inflammatory Bowel Diseases: A Systematic Review and Meta-Analysis of Randomized Controlled Trials

**DOI:** 10.1155/2013/158352

**Published:** 2013-09-24

**Authors:** Jun Ji, Yuan Lu, Huirong Liu, Hui Feng, Fuqing Zhang, Luyi Wu, Yunhua Cui, Huangan Wu

**Affiliations:** Key Laboratory for Acupuncture-Moxibustion and Immunological Effects, Shanghai University of Traditional Chinese Medicine, 650 South WanPing Road, Shanghai 200030, China

## Abstract

*Background*. Inflammatory bowel diseases (IBD) are recurrent and refractory which include ulcerative colitis (UC) and Crohn's disease (CD). Clinical researches about acupuncture and moxibustion treatments for IBD are increasing, while systematic reviews about their efficacy remains in a shortage. This study sought to evaluate the efficacy of acupuncture and moxibustion for IBD. *Methods*. Seven significant databases both in and abroad were searched for randomized controlled trials (RCTs) which compared acupuncture and moxibustion as the main intervention to pharmacotherapy in treating IBD. A meta-analysis was performed. *Results*. A total of 43 RCTs were included. Among the 43 included trials, 10 trials compared oral sulphasalazine (SASP) with acupuncture and/or moxibustion treatments. A meta-analysis of the 10 trials indicated that acupuncture and moxibustion therapy was superior to oral SASP. *Conclusion*. Acupuncture and moxibustion therapy demonstrates better efficacy than oral SASP in treating IBD. However, given the limitations of this systematic review and the included literature, definitive conclusions regarding the exact efficacy of acupuncture and moxibustion treatment for IBD cannot be drawn. Extant RCTs still cannot provide sufficient evidence and multicentre, double-blind RCTs with large sample sizes are needed to provide higher-quality evidence.

## 1. Introduction

Inflammatory bowel diseases (IBD) encompass a group of chronic nonspecific inflammatory diseases of the bowel with unknown aetiology. The main forms of IBD are ulcerative colitis (UC) and Crohn's disease (CD). UC and CD are similar with regard to their clinical manifestations, diagnosis, and treatment. Clinically, both UC and CD involve the primary symptoms of abdominal pain and diarrhoea. These conditions are refractory and recurrent, causing high levels of patient suffering. 

The aetiology and pathogenesis of IBD are not fully understood and, moreover, there is currently no specific treatment. The primary therapeutic approaches in clinical practice are derived from western medicine including traditional treatment with sulphasalazine (SASP; also known as 5-aminosalicyclic acid (5-ASA)) or the administration of steroids, immunosuppressive agents, or new biological agents. However, long-term treatment with steroids and immunosuppressants can cause serious adverse reactions, whereas new types of biological agents not only are expensive and economically burdensome to patients but also have unsatisfactory long-term efficacy. 

Since the 1990s, there have been an increasing number of clinical studies of acupuncture and moxibustion treatment for IBD, and the existing investigations have demonstrated that acupuncture and moxibustion can effectively control bowel inflammation by providing multitargeted regulation of the body's physiological balance [1, 2]. However, quality of these clinical studies is uneven, and types are varied. So far, systematic reviews or meta-analyses about acupuncture and moxibustion treatment for IBD are few. Therefore, it is necessary to conduct systematic evaluations and meta-analysis of randomized controlled trials (RCTs) of acupuncture and moxibustion in treating IBD. Also the studies could provide a reference for future clinical treatment and research. 

## 2. Materials and Methods

### 2.1. Search and Retrieval Strategy

Foreign-language and Chinese databases were searched. The Medline, Embase, and Cochrane Library databases were searched for English-language reports regarding RCTs. The Medline database was searched from 1966 to December 31, 2012, and Embase from 1974 to December 31, 2012. The Chinese literature databases that were searched include the China National Knowledge Infrastructure Database (CNKI), the Chongqing VIP Chinese Science and Technology Periodical Database (VIP), and the Chinese Biomedical Literature Database (CBM). The CNKI and CBM were searched to retrieve relevant studies from January 1, 1949, to December 31, 2012, whereas the VIP was searched to retrieve relevant studies from January 1, 1989, to December 31, 2012. The keywords used to for the Chinese-language literature include “inflammatory bowel diseases,” “ulcerative colitis”, “Crohn's disease”, “acupuncture”, “moxibustion”, “acupoint”, and “acupuncture treatment”; the keywords used for English-language literature included “acupuncture”, “moxibustion”, “inflammatory bowel diseases,” “ulcerative colitis”, and “Crohn's disease” Based on the specific circumstances of the different databases, comprehensive searches for combinations of keywords and for wildcards were conducted to ensure the completeness of the search results. 

### 2.2. Inclusion Criteria

(1) Research subjects: included studies were required to have enrolled patients with an unequivocal diagnosis of IBD (including UC and CD); no restrictions on race, age, or sex were imposed. (2) Study design: included studies were required to be RCTs in Chinese or English that evaluated the efficacy of acupuncture and/or moxibustion treatment for IBD. (3) Experimental group interventions: included studies were required to feature an experimental group that mainly received acupuncture and/or moxibustion treatment (including filiform needles, electroacupuncture, moxibustion, or cupping, among other techniques), either alone or in conjunction with other therapies (such as drug therapy), without differentiating among different acupuncture and moxibustion techniques, the selection of acupoints, or needle material. For studies in which the treatment group received acupuncture and/or moxibustion treatment combined with medication, the same drug had to be administered to both the treatment group and the control group. (4) Control group interventions: included studies were required to feature a control group that received medication, placebo, or sham acupuncture controlled treatment(s). (5) Outcome measurements: the outcome measurements of included studies had to include overall clinical efficacy, general conditions, changes in symptoms, serum inflammatory markers, and/or colonoscopic findings. (6) Availability: the full text or sufficiently informative abstracts of included studies had to be accessible.

### 2.3. Exclusion Criteria

The following types of studies were excluded from this analysis: (1) RCTs that lacked clear diagnostic criteria or basic information of the subjects or interventions; (2) serial observations, case reports, expert experiences, or descriptive analyses without control groups; (3) studies that compared different acupuncture and moxibustion techniques or selection of different acupoints to control groups; (4) studies that compared acupoint injections to drug therapy; (6) studies that were duplicate for retrieving or publishing.

### 2.4. Quality Assessments of the Included RCTs

The methodological quality of the included trials was evaluated using a modified Jadad quality scale (Table 1). The total possible score for study quality was 7 points. Studies with scores of 1–3 points were regarded as low-quality investigations, and studies with scores of 4–7 points were regarded as high-quality investigations. 

### 2.5. Data Retrieval

In accordance with the predetermined inclusion criteria, two researchers independently performed a rigorous screening to identify qualified trials, and they extracted data from these trials using a predesigned data extraction form independently. The extracted data included methodological features of the studies, demographic characteristics, treatment and control measurements, and primary outcome indicators. A third evaluator verified the consistency of the data, and any inconsistencies were addressed through discussion.

### 2.6. Data Analysis

The RevMan software package from the Cochrane Collaboration (Oxford, UK), version 5.1, was used for meta-analysis of the data. In the analysis of clinical efficacy, count data were assessed in terms of risk ratios (RRs), and continuous variables were assessed in terms of mean difference (MD). Both count data and continuous variables are expressed as efficacy values with 95% confidence intervals (CIs). If the meta-analysis results exhibited heterogeneity (defined as results of tests of heterogeneity that indicated that *P* < 0.1 and *I* ^2^ ≥ 50%), then a random effects model will be used to assess combined efficacy values; otherwise, fixed effects models will be used for these assessments. Funnel plot analysis was used to evaluate the presence of publication bias. 

## 3. Results

### 3.1. The Characteristics and Methodological Quality of the Included Trials

**Figure 1:**
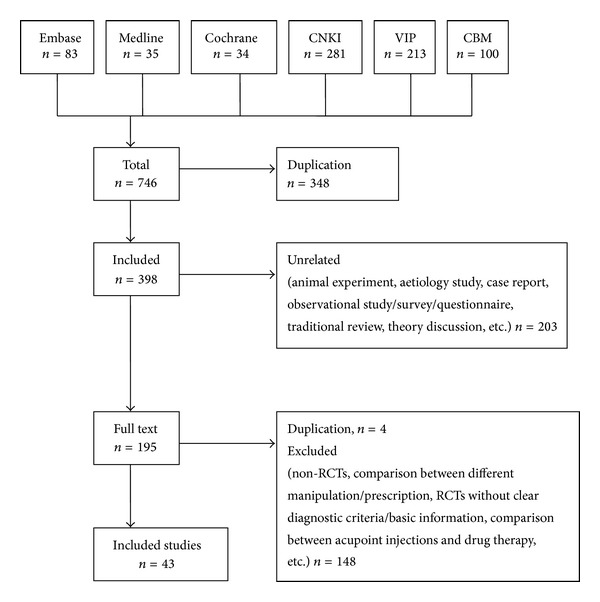
Flowchart of trial selection process. CNKI: China National Knowledge Infrastructure Database; VIP: Chongqing VIP Chinese Science and Technology Periodical Database; CBM: Chinese Biomedical Literature Database; RCT: randomized controlled trial.

Using the search and retrieval strategy, a total of 746 studies were initially retrieved from the six aforementioned medical databases, including 152 English studies and 594 Chinese studies. The bibliographic information for these studies was imported into Microsoft Excel, and 348 duplicated titles were deleted. The titles and abstracts of the remaining studies were read to exclude irrelevant studies; after this process, 195 studies remained. These 195 studies were downloaded and full texts were read; using the pre-determined exclusion criteria, 152 irrelevant studies were excluded. Finally, 43 studies were selected for inclusion [3–45], including 37 studies in Chinese and six studies in English. All of the included studies were published as journal articles. The flowchart for the literature search process is presented in Figure 1. 

Among the 43 included studies, there was one CD study [43] and 42 UC studies. These studies included a total of 4,021 patients with IBD; 2,146 of these patients were male (55.9%), 1,691 were female (44.1%), and the gender of the remaining 184 patients (4.6%) was unknown because two studies did not report this information [5, 31]. The average sample size of each included RCT was 93.5 and ranged from 29 to 640. 

Acupuncture and moxibustion therapy was the main intervention in the treatment groups of the examined RCTs. These treatments primarily involved acupuncture and/or moxibustion, although certain studies examined acupoint catgut embedding therapy, acupoint application, and auricular acupressure. In particular, among the 43 included studies, acupuncture and moxibustion therapy was used as the intervention method for the treatment group in 17 studies [5, 8, 9, 13–19, 21, 23–25, 28, 36, 45]; moxibustion treatment was used as the main intervention in 12 studies [5, 8, 9, 13–19, 28, 36]; acupuncture alone was used in one study [45]; acupoint catgut embedding therapy was used in two studies [24, 25]; balance cupping therapy was used in one study [21]; and acupoint application was used in one study [23]. Comprehensive treatment, using two forms of therapy as an intervention method, was used in 19 studies [3, 6, 7, 12, 20, 22, 26, 27, 29, 31, 33, 35, 37, 39–44]; in particular, combinations of two types of acupuncture and moxibustion treatments were used in 10 studies [3, 12, 22, 29, 35, 37, 41–44]; a combination of acupuncture and moxibustion with SASP treatments was used in three studies [6, 20, 39]; a combination of acupuncture and moxibustion with Chinese herbal treatments was used in one study [40]; and a combination of acupuncture and moxibustion with a retention enema, using traditional Chinese medicine, was used in five studies [7, 26, 27, 31, 33]. A combination of three treatments was used as an intervention method in a total of six studies [4, 10, 30, 32, 34, 38]; in particular, a combination of three types of acupuncture and moxibustion techniques was used in one study [38]; a combination of two types of acupuncture and moxibustion techniques and a retention enema using traditional Chinese medicine and/or western drugs was used in two studies [32, 34]; a combination of two types of acupuncture and moxibustion techniques with Chinese herbal medicine was used in one study [4]; and a combination therapy of two types of acupuncture and moxibustion techniques with oral western medicine was used in two studies [10, 30]. In addition, a combination of four interventions, including auricular acupressure, oral Chinese medicine, oral western medicine, and an enema with traditional Chinese medicine, was used in one study [11]. The interventions for the control groups consisted of drug therapy; in particular, most of these studies involved a control group that was administered SASP (27 studies). In addition, sham acupuncture was used as a control in two studies [41, 43]. 

The main outcome indicators reported in the included studies were overall efficacy, colonic activity indices, clinical symptom scores, fibre colonoscopy results, laboratory test findings (including evaluations of T lymphocyte subpopulations and immunoglobulin), and adverse reactions to treatments. The general data and methodological quality of the included studies are presented in Table 2, and the interventions and outcome measurements are presented in Table 3. 

The assessments for bias risk revealed that among the 43 RCTs included in this systematic evaluation, nine RCTs reported their random allocation methods [10–12, 18, 25, 29, 40, 41, 43], three studies utilized appropriate allocation concealment [10, 41, 43], and the random allocation methods and allocation concealment of the remaining trials were either inappropriate or unclear. One study reported using a single-blind approach [41], one study addressed its implementation of a single-blind design and the reasons underlying the failure of final implementation [43], and unclear descriptions of blinding were provided in two trials [30, 37]. Two studies reported the numbers and the reasons of withdrawals from the trials [41, 43]. One study utilised preliminary screening to estimate its sample size before its main experiments [41]. In general, the methodological and report qualities of the included studies were low. Four trials reported follow-up data [10, 22, 41, 43]. In particular, Zhou and Jin [10] reported that at the 1-year followup, the recurrence rate was significantly lower in the treatment group than in the control group (*P* < 0.05). Li et al. [22] observed that by the 2-year followup, 11 patients in the treatment group and 12 patients in the control group had experienced recurrences. In a 2004 CD study, Joos et al. [43] found that the efficacy was fundamentally maintained for 12 weeks of followup after treatment (*P* = 0.059), and the same research group reported in their 2006 UC study [41] that after 16-week follow-up, the primary outcome measurements remained more significantly improved than receiving treatment before (*P* < 0.001). The remaining included RCTs did not mention follow-up. 

### 3.2. Results of Studies with High Jadad Scores

Three of the 43 included trials were of high quality [10, 41, 43]. Zhou and Jin [10] utilised an RCT to observe the efficacy in UC patients of electroacupuncture combined with ginger-partitioned moxibustion and oral SASP treatment. 220 patients in this trial were randomly divided into the treatment group (*n* = 110) and the SASP control group (*n* = 110); the overall clinical efficacy of the treatment group was 84.5%, which was significantly better than the control treatment (68.2%, *P* < 0.05). In a 2004 study of CD [43] and a 2006 study of UC [41], Joos et al. examined the treatment efficacy of acupuncture combined with moxibustion, with sham acupuncture (i.e., shallow punctures at non-acupoints) as a control, in investigations that featured the rigorous design and implementation of a prospective, randomized, controlled, single-blind trial (although the researchers reported that the implementation of the single-blind design was unsuccessful in their 2004 CD study). In the 2004 study, Joos et al. randomly divided 51 CD patients into traditional Chinese medicine (TCM) group (acupuncture combined with moxibustion, *n* = 27) and control group (sham acupuncture, *n* = 24). After 4 weeks of treatment, the CD Activity Index (CDAI) of the patients in the TCM group had decreased significantly and was superior to the control group (*P* = 0.003). In their 2006 study, Joos et al. randomly divided 29 UC patients into TCM group (acupuncture combined with moxibustion, *n* = 15) and control group (sham acupuncture, *n* = 14); after 5 weeks of treatment, the Colitis Activity Index (CAI) of the patients in the traditional Chinese medicine group was significantly lower than the CAI of the control group (*P* = 0.048). 

### 3.3. Meta-Analysis Results

The 43 included RCTs featured complex interventions and different reported outcomes, with no unified efficacy standard. To develop a general understanding of the therapeutic effect of acupuncture and moxibustion therapy for IBD, intervention measurements and therapies for control group were further refined. We limited the treatment group methods to acupuncture or moxibustion alone, or a combination of acupuncture and moxibustion; this limitation produced 10 studies that compared one of the these treatments with oral SASP for the treatment of UC [5, 14–16, 18, 19, 37, 42, 44, 45]. We then conducted a comprehensive efficacy evaluation of the interventions in the 10 RCTs, which featured simple interventions that could be readily compared with oral SASP. In addition, the efficacy criteria for these RCTs were similar, featuring the three outcomes of recently cured, effective, and ineffective; judgements of these outcomes were based on various indicators, such as clinical manifestations, routine stool test results, and colonoscopy findings. The definition of ineffective treatment was consistent among these 10 studies; therefore, a meta-analysis of the overall clinical efficacies determined in these studies could be performed.

#### 3.3.1. Analysis of Overall Clinical Efficacy

**Figure 2:**
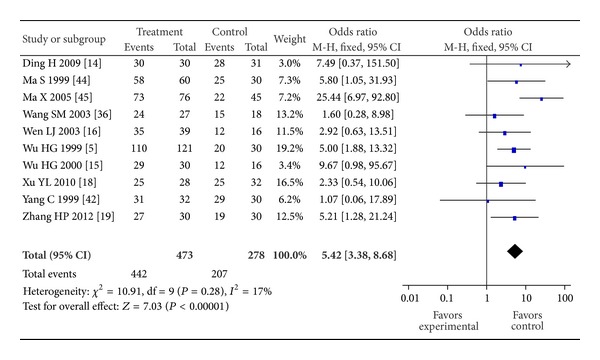
Forest plot of acupuncture and/or moxibustion for ulcerative colitis Compared to SASP.

The results of heterogeneity tests indicated that *I* ^2^ < 50% and *P* > 0.1 for the 10 examined studies and that the overall heterogeneity of subgroups was small (*P* = 0.28, *I* ^2^ = 17%); therefore, a fixed effects model was used. The overall efficacy of acupuncture alone, moxibustion alone, or acupuncture combined with moxibustion was greater than the efficacy of western medicine (oral SASP) for the treatment of IBD (*P* < 0.00001, RR = 5.42, 95% CI [3.38, 8.68]) (Figure 2).

### 3.4. Funnel Plot

**Figure 3:**
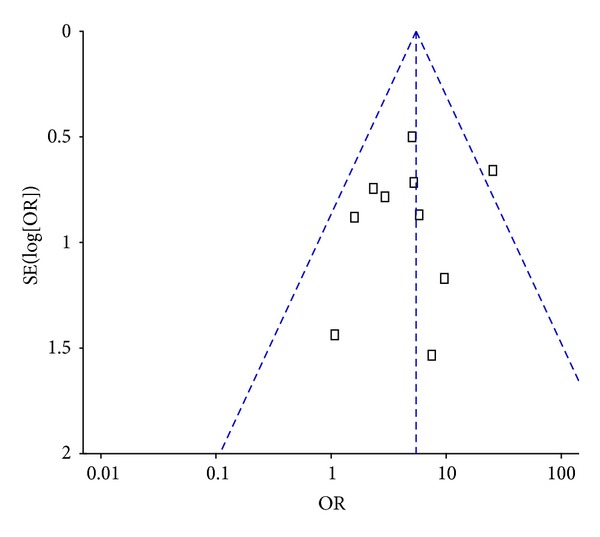
Funnel plot of randomized controlled trials using acupuncture and/or moxibustion for ulcerative colitis.

RevMan, version 5.1, was used to conduct a funnel plot analysis of the aforementioned 10 studies, and the resulting graph was symmetrical, suggesting that these studies demonstrated no obvious publication bias (Figure 3).

## 4. Discussion

### 4.1. Methodological Quality of the Included Trials

Based on the RCTs examined in this study, the methodological quality of the clinical trials regarding the examined topic was generally low, and few studies provided robust evidence. Randomization and allocation concealment are among the ways in which bias can arise, and the vast majority of the examined trials only mentioned “randomization”, without describing the specific methods used or whether allocation concealment was implemented. Thus, nonstandard “randomization” was widespread. The selective reporting of research results or the loss of trial data can also lead to reporting bias. Among the 43 included trials, only two studies reported the numbers of withdrawals from the study and the reasons; because none of the other trials reported exit data or cases lost to followup, the efficacy conclusions of these trials might be exaggerated. Most of the studies did not utilize blinding, producing a high probability of bias. The overall quality of the studies was low, affecting the strength of the evidence that was examined in this systematic evaluation. 

### 4.2. Determinations of Sample Size

Adequate attention must be devoted to the important factor of sample size in RCTs that address the examined topic. At present, only two relevant RCTs have featured sample sizes of more than 200 individuals. Insufficient sample sizes can reduce the power of a test, resulting in limited reliability of the results and conclusions, that do not truly reflect the overall effects observed in a study. Low statistical power will reduce the magnitude of evidence that RCTs can provide. 

### 4.3. Selection of Interventions

In this study, the specific interventions described in the included studies, which included acupuncture, herb-partitioned moxibustion, ginger-partitioned moxibustion, moxibustion, electroacupuncture, abdominal acupuncture, acupuncture catgut embedding, acupoint application, cupping, and auricular pressure, were treated as one type of therapy, without considering the differences in acupoint selection or therapeutic techniques. Therefore, the results of this study might indicate overall efficacy trends, but they cannot be utilised to draw definitive conclusions, thus limiting the extent to which the conclusions of this investigation can be applied.

### 4.4. The Selection and Measurement of Outcome Indicators

The majority of the included studies selected clinical efficacy as the outcome measurement. Thus, there may exist subjectivity in the evaluations of the results. With little use of objective indicators, such as clinical symptom scores, endoscopic scores, or pathologic scores, the tendency towards subjective judgements weakened the credibility of conclusions regarding the effectiveness of acupuncture and moxibustion. With regard to long-term efficacy, most of the included studies did not conduct long-term followup, or if someone did, either the data for withdraw or the follow-up methods were not described. As a result, the long-term efficacy of acupuncture and moxibustion treatment for IBD cannot be determined. 

### 4.5. Suggestions for Future Research

The objectivity and accuracy of systematic evaluations rely on high-quality RCTs. The findings of this systematic evaluation are somewhat limited due to the generally low quality of the existing studies. Thus, it is recommended that future research should be based on the Consolidated Standards of Reporting Trials (CONSORT) statement [46]. In particular, future studies should provide detailed reports regarding the generation of random allocation sequences and allocation concealment; moreover, to the greatest possible extent, these studies should be blinded and placebo controlled. In addition, a subject flowchart should be utilised to provide detailed accounts of patient withdrawals and loss during trials. Followup should be strengthened, and all of the trial data should be completely reported. In addition, intent-to-treat analysis should be conducted to evaluate therapeutic effects. These measurements will produce clinical RCTs that provide high levels of reliable evidence. 

## 5. Conclusion

The results of this study suggest that acupuncture and moxibustion treatment demonstrated better overall efficacy than oral SASP in treating IBD. However, given the limitations of this systematic evaluation and the included studies, definitive conclusions cannot be drawn with regard to the specific efficacy of acupuncture and moxibustion treatment for IBD. Currently published RCTs have not provided sufficient evidence for the effectiveness of acupuncture and moxibustion for IBD; thus, multicentre, double-blind RCTs with large sample sizes are still required to provide higher levels of evidence. 

## Figures and Tables

**Table 1:** Modified Jadad quality scale.

Aspects	Details	Score
Randomization	Appropriate if random sequence is generated by computer or similar methods	2
	Unclear if a trial does not describe its method of randomization	1
	Inappropriate if a study uses an alternate assignment method, such as the allocation of odd and even numbers	0

Randomization concealment	Appropriate if either the distribution scheme is controlled by a center or pharmacy, containers with consistent serial numbers being used, on-site computer control, sealed opaque envelopes, or any other allocation method that clinicians and subjects are unable to predict	2
	Unclear if only a random number table or other random allocation scheme is employed	1
	Inappropriate if either of alternate allocation, case numbers, days of the week, an open-label random number table, serial coded envelopes, or any other method with predictable assignments is used	0
	Absent if randomization is not used	0

Blinding method	Appropriate if a completely identical placebo form or a similar method is used	2
	Unclear if the trial was described as blinded, but no methodological information regarding the blinding was provided	1
	Inappropriate if the double-blind method is not adopted or if the blinding method is improper, such as a comparison between tablets and injections	0

Withdrawal and exit	The number and reasons of patients who withdraw or exit are described	1
	The number and reasons of patients who withdraw or exit are not described	0

**Table 2:** Characteristics and methodological quality of included studies.

Study	Sample size (*n*_1_/*n*_2_)	Sex (male/female)	Age (mean or range)	Type of IBD	Followup	Methodology quality score
Ma and Zhang, 1997 [3]	90 (60/30)	56/34	*T*: 23–68; *C*: 28–65	UC	No	1
Gao, 1997 [4]	66 (46/20)	27/39	*T*: 43.6; *C*: 39.5	UC	No	1
Wu et al., 1999 [5]	151 (65/56/30)	ND	*T*: 42.7; *C*: 38.4	UC	No	1
Li et al., 2008 [6]	67 (34/33)	42/25	35.5	UC	No	1
Mo et al., 2010 [7]	62 (31/31)	35/27	35.5	UC	No	1
X. Guo and F. Guo, 2010 [8]	55 (28/27)	38/17	38.77	UC	No	1
Yang et al., 2011 [9]	100 (50/50)	61/39	*T*: 54.6; *C*: 55.3	UC	No	1
Zhou and Jin, 2008 [10]	220 (110/110)	131/89	*T*: 48.60; *C*: 50.24	UC	Yes	4
Han et al., 2012 [11]	81 (41/40)	47/34	*T*: 36.5; *C*: 34.7	UC	No	1
Jiang, 2012 [12]	80 (40/40)	39/41	*T*: 38.65; *C*: 39.35	UC	No	3
Zhou, 2003 [13]	66 (34/32)	31/35	40.8	UC	No	1
Din et al., 2009 [14]	61 (30/31)	32/29	*T*: 44.9; *C*: 40.2	UC	No	1
Wu et al., 2000 [15]	46 (30/16)	25/21	*T*: 38.75; *C*: 37	UC	No	1
Wen, 2003 [16]	69 (39/30)	35/34	*T*: 41.2; *C*: 37.4	UC	No	1
Wang et al., 2006 [17]	60 (30/30)	28/32	38.5	UC	No	1
Xu et al., 2010 [18]	60 (28/32)	35/25	*T*: 35.0; *C*: 37.0	UC	No	2
Zhang, 2012 [19]	60 (30/30)	32/28	*T*: 28–52; *C*: 27–55	UC	No	1
Chi and Yu, 2011 [20]	84 (44/40)	36/48	*T*: 45; *C*: 43	UC	No	1
Luo, 2009 [21]	76 (40/36)	42/34	*T*: 53; *C*: 51.3	UC	No	1
Li et al., 2006 [22]	68 (40/28)	40/28	*T*: 36.4; *C*: 38.2	UC	Yes	1
Tian et al., 2012 [23]	106 (53/53)	46/60	*T*: 29–61; *C*: 32–63	UC	No	1
Chen, 2004 [24]	130 (100/30)	80/50	*T*: 42.5; *C*: 40.2	UC	No	1
Li et al., 2006 [25]	116 (56/60)	52/64	*T*: 37.1; *C*: 37.3	UC	No	2
Duan et al., 2012 [26]	640 (320/320)	406/234	*T*: 45.5; *C*: 46.5	UC	No	1
Sun and Wang, 1998 [27]	88 (45/43)	43/45	*T*: 34.6; *C*: 33.8	UC	No	1
Wang, 2008 [28]	108 (54/54)	78/30	*T*: 35; *C*: 33.5	UC	No	1
Ma and Xu, 2005 [29]	92 (47/45)	51/41	*T*: 52.5; *C*: 52.5	UC	No	2
Cui, 2010 [30]	48 (24/24)	27/21	*T*: 43; *C*: 44	UC	No	2
Guo et al., 2007 [31]	33 (22/11)	ND	ND	UC	No	1
Wang et al., 2009 [32]	78 (39/39)	44/34	*T*: 57.5; *C*: 55.0	UC	No	1
Long and Yang, 2010 [33]	46 (23/23)	20/26	*T*: 33.5; *C*: 33.4	UC	No	1
Chen, 2010 [34]	168 (84/84)	91/77	38.4	UC	No	1
Sun and Wang, 2001 [35]	55 (35/20)	32/23	*T*: 22–65; *C*: 25–70	UC	No	1
Wang et al., 2006 [36]	45 (27/18)	23/22	40.5	UC	No	1
Shi et al., 2006 [37]	60 (30/30)	34/26	*T*: 42.31; *C*: 43.64	UC	No	2
Qun et al., 2012 [38]	63 (33/30)	32/31	*T*: 46; *C*: 41	UC	No	1
Xu, 2006 [39]	110 (56/54)	59/51	*T*: 35.5; *C*: 33	UC	No	1
Zhang et al., 2011 [40]	60 (30/30)	38/22	*T*: 31.2; *C*: 30.6	UC	No	2
Joos et al., 2006 [41]	29 (15/14)	10/19	37.89 ± 12.0	UC	Yes	7
Yang and Yan, 1999 [42]	62 (32/30)	30/32	*T*: 45.5; *C*: 50.1	UC	No	1
Joos et al., 2004 [43]	51 (27/24)	15/36	ND	CD	Yes	5
Ma, 1999 [44]	90 (60/30)	56/34	*T*: 23–68; *C*: 28–65	UC	No	1
Ma, 2005 [45]	121 (76/45)	67/54	*T*: 42; *C*: 41.5	UC	No	1

*n*_1_: sample size of test group; *n*_2_: sample size of control group; *T*: test group; *C*: control group; ND: not described; IBD: inflammatory bowel disease; UC: ulcerative colitis; CD: Crohn's disease.

**Table 3:** Interventions and outcomes of included studies.

Study	Intervention	Control	Outcome measurement
Ma and Zhang, 1997 [3]	Acupuncture + sparrow-pecking moxibustion	SASP + metronidazole	Efficacy
Gao, 1997 [4]	Acupoint application + moxibustion + decoction of traditional Chinese medicine	Traditional Chinese medicine	Efficacy
Wu et al., 1999 [5]	Drug-separated moxibustion	SASP	Efficacy, T lymphocyte subpopulations, HLA-DR antigen
Li et al., 2008 [6]	Moxa-box moxibustion + SASP	SASP	Efficacy, haemorheology, immunoglobulin, T lymphocyte subpopulations
Mo et al., 2010 [7]	Moxa-box moxibustion + traditional Chinese medicine enema	Traditional Chinese medicine enema	Efficacy
X. Guo and F. Guo, 2010 [8]	Warm moxibustion of acupoints	SASP + PAT	Efficacy, T lymphocyte subpopulations
Yang et al., 2011 [9]	Ginger moxibustion	Diphenoxylate	Efficacy
Zhou and Jin, 2008 [10]	Electroacupuncture + ginger moxibustion + SASP	SASP	Medical condition, efficacy, intestinal mucosa pathology, adverse reactions
Han et al., 2012 [11]	Auricular acupressure + salicylic acid preparations + traditional Chinese medicine + traditional Chinese medicine enema	Salicylic acid preparations + traditional Chinese medicine + traditional Chinese medicine enema	Enema retention time, efficacy
Jiang, 2012 [12]	Abdominal acupuncture + acupoint catgut embedding	Bupi Yichang pills	Symptom scores, efficacy, fibre colonoscopy
Zhou, 2003 [13]	Ginger moxibustion	SASP + prednisone tablets	Efficacy
Din et al., 2009 [14]	Ginger moxibustion	SASP	Efficacy
Wu et al., 2000 [15]	Moxibustion with herbal medicine underneath	SASP	Efficacy, colonic mucosal histopathology, mucin
Wen, 2003 [16]	Drug-separated moxibustion	SASP	Efficacy, immunoglobulin
Wang et al., 2006 [17]	Drug-separated moxibustion	SASP + metronidazole tablets	Efficacy, immunoglobulin, T lymphocyte subpopulations, NK content
Xu et al., 2010 [18]	Herb-partitioned moxibustion	SASP	Efficacy, adverse reactions
Zhang, 2012 [19]	Drug-separated moxibustion	SASP	Efficacy
Chi and Yu, 2011 [20]	Umbilical compression with traditional Chinese medicine + SASP	SASP	Efficacy
Luo, 2009 [21]	Balance cupping	Enteritidis tablet	Efficacy, symptom scores, immunoglobulin
Li et al., 2006 [22]	Application of musky warm umbilical cream + use of a specific electromagnetic spectrum therapeutic apparatus	SASP	Efficacy
Tian et al., 2012 [23]	Acupoint application	SASP	Efficacy, symptom scores
Chen, 2004 [24]	Acupoint catgut embedding	SASP	Efficacy
Li et al., 2006 [25]	Acupoint catgut embedding	SASP	Efficacy, stool characteristics, abdominal pain
Duan et al., 2012 [26]	Acupoint catgut embedding + traditional Chinese medicine enema	Traditional Chinese medicine enema	Efficacy, routine stool tests
Sun and Wang, 1998 [27]	Warm acupuncture + traditional Chinese medicine enema	Traditional Chinese medicine enema	Efficacy
Wang, 2008 [28]	Warm acupuncture	SASP + western medicine enema	Efficacy
Ma and Xu, 2005 [29]	Acupuncture + TDP	SASP	Efficacy
Cui, 2010 [30]	Acupuncture + moxibustion + SASP	SASP	Efficacy, serum levels of TNF-*α*, IL-1, and IL-10
Guo et al., 2007 [31]	Acupuncture + traditional Chinese medicine enema	Traditional Chinese medicine enema	Efficacy, intestinal microscopy
Wang et al., 2009 [32]	Electroacupuncture + ginger moxibustion + traditional Chinese and western medicine enema	Traditional Chinese and western medicine enema	Efficacy
Long and Yang, 2010 [33]	Acupuncture + traditional Chinese medicine enema	Traditional Chinese medicine enema	Efficacy, colonoscopy
Chen, 2010 [34]	Acupuncture + ginger moxibustion + traditional Chinese medicine enema	Traditional Chinese medicine enema	Efficacy
Sun and Wang, 2001 [35]	Acupuncture + ginger moxibustion	Shuanghuanglian compound + norfloxacin + gentamicin	Efficacy
Wang et al., 2006 [36]	Warm acupuncture	SASP	Efficacy
Shi et al., 2006 [37]	Electroacupuncture + moxibustion	SASP	Efficacy, serum levels of TNF-*α*, IL-8, IL-l, and IL-10
Qun et al., 2012 [38]	Acupuncture + moxibustion + TDP	SASP	Efficacy, intestinal mucosa pathology, immunoglobulin
Xu, 2006 [39]	Ginger moxibustion + SASP	SASP	Efficacy
Zhang et al., 2011 [40]	Traditional acupuncture + Chinese medicine decoction	SASP	Efficacy, symptom scores, serum levels of IL-8 and IL-10
Joos et al., 2006 [41]	Traditional acupuncture + moxibustion	Sham acupuncture	CAI, QLO, general well-being, C-reactive protein, serum *α*_1_-acid glycoprotein
Yang and Yan, 1999 [42]	Acupuncture + moxibustion	SASP	Efficacy, routine examination of faeces, electrogastrograms, sigmoidoscopy
Joos et al., 2004 [43]	Traditional acupuncture + moxibustion	Sham acupuncture	CDAI, QLO, general condition, C-reactive protein, serum *α*_1_-acid glycoprotein
Ma, 1999 [44]	Acupuncture + sparrow-pecking moxibustion	SASP	Efficacy
Ma, 2005 [45]	Acupuncture	SASP	Efficacy

SASP: sulphasalazine; IBDQ: Inflammatory Bowel Disease Questionnaire; CAI: Colitis Activity Index; QOL: quality of life; CDAI: Crohn's Disease Activity Index; PAT: pipemidic acid tablet; sham acupuncture: superficial needling at nonacupoints.
